# Dibarium tricadmium bis­muthide(-I,-III) oxide, Ba_2_Cd_3−δ_Bi_3_O

**DOI:** 10.1107/S1600536810046283

**Published:** 2010-11-17

**Authors:** Sheng-Qing Xia, Svilen Bobev

**Affiliations:** a Department of Chemistry and Biochemistry, University of Delaware, Newark, DE 19716, USA; b State Key Laboratory of Crystal Materials, Institute of Crystal Materials, Shandong University, Jinan, Shandong 250100, People’s Republic of China

## Abstract

Ba_2_Cd_2.13_Bi_3_O, a new bis­muthide(-I,-III) oxide, crystallizes with a novel body-centered tetra­gonal structure (Pearson code *tI*36). The crystal structure contains eight crystallographically unique sites in the asymmetric unit, all on special positions. Two Ba, one Cd and two Bi atoms have site symmetry 4*mm*, the third Bi atom has *mmm*. and the O atom has 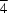 *m*2 symmetry; the second Cd site (2*mm*. symmetry) is not fully occupied. The layered structure is complex and can be considered as an inter­growth of two types of slabs, *viz.* BaCdBiO with the ZrCuSiAs type and BaCd_2_Bi_2_ with the CeMg_2_Si_2_ type.

## Related literature

Isotypic compounds are not known; however, there are several compounds whose structures are based on fused CdBi_4_ tetra­hedral fragments, including BaCdBi_2_ (Brechtel *et al.*, 1981 ▶), Ba_11_Cd_8_Bi_14_ (Xia & Bobev, 2006*a* ▶), Eu_10_Cd_8_Bi_12_ (Xia & Bobev, 2007 ▶), Sr_21_Cd_4_Bi_18_ (Xia & Bobev, 2008 ▶). Condensed trigonal CdBi_5_ bi-pyramids and distorted CdBi_6_ octa­hedra are known for Ba_2_Cd_3_Bi_4_ (Cordier *et al.*, 1982 ▶; Xia & Bobev, 2006*b* ▶). The serendipitous discovery of the title compound was the result of a systematic study of the Ba—Cd—Bi system, inspired from the identification of Ba_3_Cd_2_Sb_4_ (Saparov *et al.*, 2008 ▶). The compound BaCdSbF (Saparov & Bobev, 2010 ▶) is an example of a structure that epitomizes the BaCdBiO slabs. Recently, the idea that inter­metallic oxide-pnictides and fluoride-pnictides could be a widespread class of quaternary solids has been discussed on the examples of Ba_5_Cd_2_Sb_5_O_*x*_ (0.5<*x*<0.7) and Ba_5_Cd_2_Sb_5_F (Saparov & Bobev, 2010 ▶). Theoretical considerations of non-classical electron-rich networks of the pnictogen elements is proved by Papoian & Hoffmann (2000 ▶). For standardization of the atomic coord­in­ates, the program *STRUCTURE-TIDY* was used (Gelato & Parthé, 1987) ▶. For further information on structure types among inter­metallic phases, we refer to Pearson’s Handbook (Villars & Calvert, 1991 ▶).

## Experimental

### 

#### Crystal data

Ba_2_Cd_2.13_Bi_3_O
*M* *_r_* = 1148.47
Tetragonal, 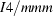

*a* = 4.7396 (4) Å
*c* = 43.601 (7) Å
*V* = 979.5 (2) Å^3^
*Z* = 4
Mo *K*α radiation
μ = 66.05 mm^−1^
*T* = 120 K
0.05 × 0.05 × 0.02 mm

#### Data collection

Bruker SMART APEX diffractometer
Absorption correction: multi-scan (*SADABS*; Bruker, 2002 ▶) *T*_min_ = 0.137, *T*_max_ = 0.352
5274 measured reflections
433 independent reflections
386 reflections with *I* > 2σ(*I*)
*R*_int_ = 0.066

#### Refinement

*R*[*F* ^2^ > 2σ(*F* ^2^)] = 0.032
*wR*(*F* ^2^) = 0.073
*S* = 1.22
433 reflections
25 parameters
Δρ_max_ = 4.75 e Å^−3^
Δρ_min_ = −1.93 e Å^−3^

### 

Data collection: *SMART* (Bruker, 2002 ▶); cell refinement: *SAINT* (Bruker, 2002 ▶); data reduction: *SAINT*; program(s) used to solve structure: *SHELXTL* (Sheldrick, 2008) ▶; program(s) used to refine structure: *SHELXTL*; molecular graphics: *XP* in *SHELXTL* and *CrystalMaker* (*CrystalMaker*, 2009 ▶); software used to prepare mat­erial for publication: *SHELXTL*.

## Supplementary Material

Crystal structure: contains datablocks I, global. DOI: 10.1107/S1600536810046283/wm2421sup1.cif

Structure factors: contains datablocks I. DOI: 10.1107/S1600536810046283/wm2421Isup2.hkl

Additional supplementary materials:  crystallographic information; 3D view; checkCIF report

## Figures and Tables

**Fig. 1.**
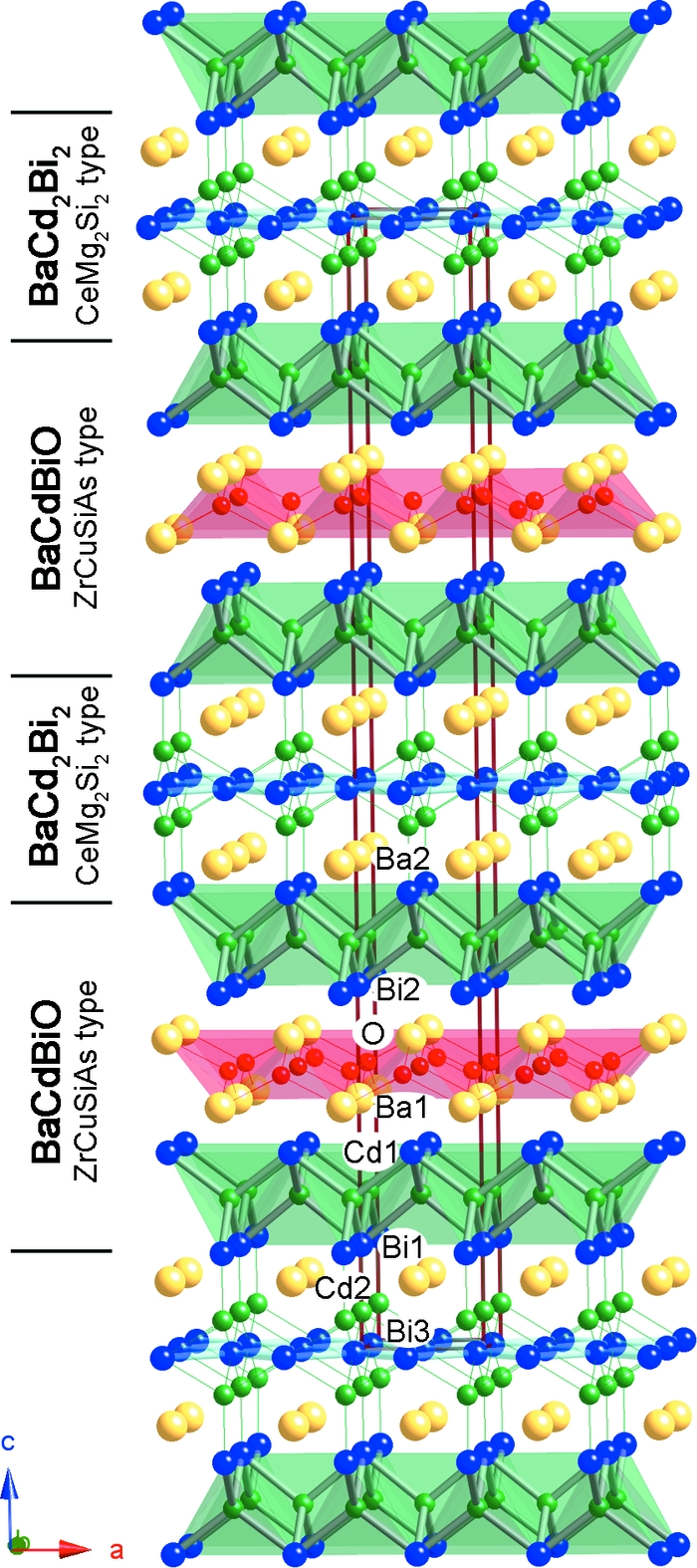
Combined ball-and-stick and polyhedral representation of the crystal structure of the tetragonal Ba2Cd2.13Bi3O, viewed approximately along [010]. The unit cell is outlined. Color code: Ba - light yellow, Cd - green, Bi - blue, and O - red.

**Fig. 2.**
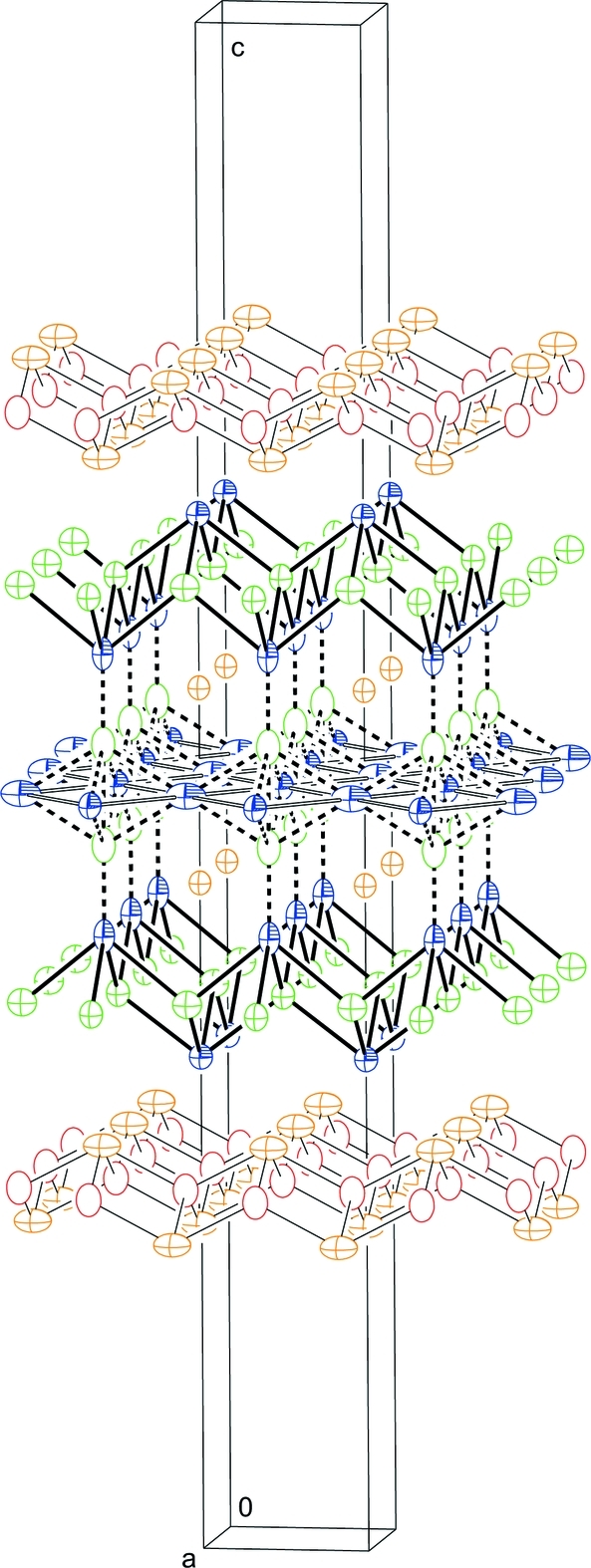
A plot of the Ba2Cd2.13Bi3O structure with displacement ellipsoids drawn at the 95% probability level. Color code: Ba - light yellow, Cd - green, Bi - blue, and O - red. Cd2, which is partially occupied, is connected to the neighboring Bi atoms with broken cylinders. The long Bi3–Bi3 bonds within the square nest are depicted as open cylinders, while the mostly ionic Ba–O interactions are represented with thin lines. The unit cell is outlined.

## supplementary crystallographic information

### Comment

Our previous work in the *A*–Cd–Bi systems, where the symbol '*A*' is used to denote Ca, Sr, Ba, Eu, and Yb, led to the identification of several novel compounds such as Ba_11_Cd_8_Bi_14_ (Xia & Bobev, 2006*a*), Eu_10_Cd_8_Bi_12_ (Xia & Bobev, 2007), Sr_21_Cd_4_Bi_18_ (Xia & Bobev, 2008), among others. During these exploratory investigations, a new phase was serendipitously discovered. Upon subsequent structural work by means of single-crystal X-ray diffraction, it turned out to be the quaternary bismuthide(-I,-III) oxide Ba_2_Cd_2.13_Bi_3_O. It crystallizes in space group *I*4/*mmm* in what appears to be a structure with a previously unreported structure type.

The crystal structure of the title compound is shown schematically in Figure 1.  In this representation, the layered nature of the structure and the basic building blocks are emphasized.  As seen from the plot, it can be readily described as consisting of PbO-type layers of fused [CdBi_4_] tetrahedra, running parallel to the *ab* plane and which are alternately stacked along the *c* axis with BaO slabs and Bi square-nets (Figure 1). The actual structure is more complicated due to the partially occupied Cd2 site. The Cd2 atoms cap the Bi square-nets from above and below and link these fragments to the CdBi slabs. Figure 2 shows a representation with anisotropic displacement ellipsoids.

The observed Cd–Bi (from 2.9688 (14) to 3.0565 (14) Å) and Bi–Bi distances (3.3514 (3) Å)  are comparable to those reported for other cadmium-bismuthides such as BaCdBi_2_ (Brechtel *et al.*, 1981), Ba_11_Cd_8_Bi_14_ (Xia & Bobev, 2006*a*), Eu_10_Cd_8_Bi_12_ (Xia & Bobev, 2007), Sr_21_Cd_4_Bi_18_ (Xia & Bobev, 2008), Ba_2_Cd_3_Bi_4_ (Cordier *et al.*, 1982; Xia & Bobev, 2006*b*). The Cd–Bi distances involving the Cd2 atoms are shorter, but due to the very low occupancy of the Cd site (close to 1/8 occupied), the physical significance of such contacts is hard to be rationalized.  The Ba–O contacts (2.6736 (14) Å) match well the recently reported Ba–O distances for Ba_5_Cd_2_Sb_5_O*_x_* (0.5<*x*<0.7) (Saparov & Bobev, 2010).

Being a new structure type, it is important to relate the structure of the title compound to the structure(s) of previously reported phases with known structure types (Villars & Calvert, 1991). A good starting point for a discussion is BaCdBi_2_ (Brechtel *et al.*, 1981), reported with the ZrAl_3_ type (Villars & Calvert, 1991).  Coincidentally, BaCdBi_2_ also crystallizes in space group *I*4/*mmm* and with cell parameters *a* = 4.77 Å and *c* = 23.6 Å.  This structure features the very same PbO-type CdBi layers, stacked along the *c*-axis in alternating order with Bi square-nets.  Not considering the partially occupied Cd2 site (for simplicity), one can then immediately reason that replacing every other BaBi slab in BaCdBi_2_ with a BaO slab will yield a hypothetical Ba_2_Cd_2_Bi_3_O compound. The latter can be considered as a super-structure of BaCdBi_2_ with doubled periodicity along the stacking detection, i.e., the *c* axis.  Another way to relate the structure under consideration to other structure types is to consider the Cd2 site fully occupied and rationalize the structure of such an ordered Ba_2_Cd_3_Bi_3_O compound as an intergrowth of two types of slabs – BaCdBiO with the ZrCuSiAs type and BaCd_2_Bi_2_ with the CeMg_2_Si_2_ type, respectively. This line of thinking is schematically illustrated in Figure 1.

### Experimental

Handling of the reagents was done in an argon-filled glove box or under vacuum. All metals were with a stated purity higher than 99.9% (metal basis). They were purchased from Alfa, kept in a glove box, and were used as received.

The flux reaction was carried out in a 2 cm^3^ alumina crucible, using a mixture of elemental Ba and Cd in a molar ratio 3 : 2 and *ca* 2.1 grams of Bi. The reaction was aimed at growing crystals of Ba_3_Cd_2_Bi_4_, a hitherto unknown phase with the Ba_3_Cd_2_Sb_4_ structure (Saparov *et al.*, 2008), using excess of bismuth as a metal flux. The crucible was subsequently enclosed and flame-sealed in an evacuated fused silica ampoule, and then was heated at 200Kh^-1^ to 973 K, homogenized at 973 K for 20 h, cooled at a rate of -5Kh^-1^ to 723 K, where the excess Bi was removed by decanting it, leaving behind some irregularly shaped silver pieces and a few dark-to-black plates. The former were confirmed (via single-crystal and powder X-ray diffraction) to be Ba_2_Cd_3_Bi_4_ (Xia & Bobev, 2006*b*) and the latter turned out to be the title compound.

After the structure of the new compound was solved from single-crystal X-ray diffraction data, it was realized that an unadventurous exposure of the starting materials to air has led to the formation of Ba_2_Cd_2.13_Bi_3_O (minor product), alongside the intermetallic phase (major product). Subsequent attempts to produce Ba_2_Cd_2.13_Bi_3_O in quantitative yields from reactions of Ba, Cd, Bi and BaO_2_ (Acros, 95%) were not successful, suggesting it might be a metastable phase.

### Refinement

The observed reflections satisfied the systematic extinction conditions for a body-centered cell, and the centrosymmetric space group *I*4/*mmm* (No. 139) was chosen based on intensity statistics. The structure was successfully solved by direct methods, which located six atomic positions – the two alkaline-earth metals, the three Bi atoms and one Cd atom. Subsequent structure refinements by full matrix least-squares methods on F^2^ showed the location of the oxygen atom in a tetrahedral void of Ba atoms with Ba–O distances of 2.6736 (14) Å. The difference Fourier map, however, also showed a residual peak of about 15 e^-^ Å^-3^, located ca. 2.7 Å away from Bi. At first, we attempted to refine this as oxygen, however, there were serious problems with this model: 1) the electron density was much higher than a fully occupied O^2-^; 2) such coordination is inconsistent with the bonding requirements of oxygen; 3) the electron count was clearly implausible, *viz*. (Ba^2+^)_2_(Cd^2+^)_2_(Bi^3-^)_2_(Bi^1-^)(O^2-^)_2_. Here, the polyanionic networks features bismuth in two different coordination modes, which require different formal charges. The Bi atoms in the square-net are hypervalent, thus formally Bi^1-^, as analyzed computationally elsewhere (Papoian & Hoffmann, 2000). Therefore, this additional site was modeled as a partially occupied Cd atom (Cd2). The formal electron count taking into account the ca. 1/8 occupied Cd2 site is then (Ba^2+^)_2_(Cd^2+^)_2.13_(Bi^3-^)_2_(Bi^1-^)(O^2-^), rendering this model much more reasonable (despite the shortcoming of the shorter Cd2–Bi distances, vide supra)

The occupancy of Cd2 was fixed at 12.5%. After including the partially occupied Cd2 site, the refinement converged at low residuals, accompanied with a flat final difference Fourier map - the maximum residual electron density lies 0.74 Å from Bi1, and the minimum residual electron density lies 2.33 Å from O.

In the final refinement cycles, all atoms were refined with anisotropic displacement parameters and with coordinates standardized using the software STRUCTURE-TIDY (Gelato & Parthε, 1987).

### Crystal data

**Table:**

Ba_2_Cd_2.13_Bi_3_O	*D*_x_ = 7.788 Mg m^−^^3^
*M**_r_* = 1148.47	Mo *K*α radiation, λ = 0.71073 Å
Tetragonal, *I*4/*m**m**m*	Cell parameters from 938 reflections
Hall symbol: -I 4 2	θ = 4.7–26.7°
*a* = 4.7396 (4) Å	µ = 66.05 mm^−^^1^
*c* = 43.601 (7) Å	*T* = 120 K
*V* = 979.5 (2) Å^3^	Plate, black
*Z* = 4	0.05 × 0.05 × 0.02 mm
*F*(000) = 1890	

### Data collection

**Table:**

Bruker SMART APEX diffractometer	433 independent reflections
Radiation source: fine-focus sealed tube	386 reflections with *I* > 2σ(*I*)
graphite	*R*_int_ = 0.066
ω scans	θ_max_ = 28.2°, θ_min_ = 0.9°
Absorption correction: multi-scan (*SADABS*; Bruker, 2002)	*h* = −6→6
*T*_min_ = 0.137, *T*_max_ = 0.352	*k* = −6→6
5274 measured reflections	*l* = −56→56

### Refinement

**Table:**

Refinement on *F*^2^	0 restraints
Least-squares matrix: full	Primary atom site location: structure-invariant direct methods
*R*[*F*^2^ > 2σ(*F*^2^)] = 0.032	Secondary atom site location: difference Fourier map
*wR*(*F*^2^) = 0.073	*w* = 1/[σ^2^(*F*_o_^2^) + (0.0055*P*)^2^ + 124.164*P*] where *P* = (*F*_o_^2^ + 2*F*_c_^2^)/3
*S* = 1.22	(Δ/σ)_max_ < 0.001
433 reflections	Δρ_max_ = 4.75 e Å^−^^3^
25 parameters	Δρ_min_ = −1.93 e Å^−^^3^

### Special details

**Table:**

Experimental. Selected in the glove box, crystals were put in a Paratone N oil and cut to the desired dimensions. The chosen crystal was mounted on a tip of a glass fiber and quickly transferred onto the goniometer. The crystal was kept under a cold nitrogen stream to protect from the ambient air and moisture.Data collection is performed with four batch runs at φ = 0.00 ° (607 frames), at φ = 90.00 ° (607 frames), at φ = 180.00 ° (607 frames), and at φ = 270.00 (607 frames). Frame width = 0.30 ° in ω. Data are merged and treated with multi-scan absorption corrections.
Geometry. All esds (except the esd in the dihedral angle between two l.s. planes) are estimated using the full covariance matrix. The cell esds are taken into account individually in the estimation of esds in distances, angles and torsion angles; correlations between esds in cell parameters are only used when they are defined by crystal symmetry. An approximate (isotropic) treatment of cell esds is used for estimating esds involving l.s. planes.
Refinement. Refinement of *F*^2^ against ALL reflections. The weighted *R*-factor wR and goodness of fit *S* are based on *F*^2^, conventional *R*-factors *R* are based on *F*, with *F* set to zero for negative *F*^2^. The threshold expression of *F*^2^ > σ(*F*^2^) is used only for calculating *R*-factors(gt) etc. and is not relevant to the choice of reflections for refinement. *R*-factors based on *F*^2^ are statistically about twice as large as those based on *F*, and *R*- factors based on ALL data will be even larger.

### Fractional atomic coordinates and isotropic or equivalent isotropic displacement parameters (Å^2^)

**Table:**

	*x*	*y*	*z*	*U*_iso_*/*U*_eq_	Occ. (<1)
Ba1	0.0000	0.0000	0.22161 (7)	0.0529 (9)	
Ba2	0.0000	0.0000	0.43606 (5)	0.0129 (4)	
Cd1	0.0000	0.5000	0.13679 (4)	0.0181 (4)	
Cd2	0.0000	0.0000	0.0330 (6)	0.020 (4)	0.13
Bi1	0.0000	0.0000	0.09251 (3)	0.0165 (3)	
Bi2	0.0000	0.0000	0.32220 (3)	0.0145 (3)	
Bi3	0.0000	0.5000	0.0000	0.0237 (4)	
O	0.0000	0.5000	0.2500	0.037 (7)	

### Atomic displacement parameters (Å^2^)

**Table:**

	*U*^11^	*U*^22^	*U*^33^	*U*^12^	*U*^13^	*U*^23^
Ba1	0.0665 (15)	0.0665 (15)	0.0257 (14)	0.000	0.000	0.000
Ba2	0.0120 (6)	0.0120 (6)	0.0148 (10)	0.000	0.000	0.000
Cd1	0.0133 (9)	0.0217 (10)	0.0193 (9)	0.000	0.000	0.000
Cd2	0.014 (6)	0.014 (6)	0.032 (12)	0.000	0.000	0.000
Bi1	0.0109 (4)	0.0109 (4)	0.0278 (7)	0.000	0.000	0.000
Bi2	0.0137 (4)	0.0137 (4)	0.0161 (6)	0.000	0.000	0.000
Bi3	0.0123 (7)	0.0415 (9)	0.0173 (7)	0.000	0.000	0.000
O	0.029 (10)	0.029 (10)	0.052 (19)	0.000	0.000	0.000

### Geometric parameters (Å, °)

**Table:**

Ba1—O^i^	2.6736 (14)	Cd2—Cd2^xv^	2.87 (5)
Ba1—O	2.6736 (14)	Cd2—Ba2^i^	3.613 (9)
Ba1—O^ii^	2.6736 (14)	Cd2—Ba2^iv^	3.613 (9)
Ba1—O^iii^	2.6736 (14)	Cd2—Ba2^v^	3.613 (9)
Ba1—Bi2^iv^	3.8575 (16)	Cd2—Ba2^ii^	3.613 (9)
Ba1—Bi2^i^	3.8575 (16)	Bi1—Cd1^xiii^	3.0565 (14)
Ba1—Bi2^ii^	3.8575 (16)	Bi1—Cd1^xi^	3.0565 (14)
Ba1—Bi2^v^	3.8575 (16)	Bi1—Cd1^iii^	3.0565 (14)
Ba2—Bi1^iv^	3.5756 (9)	Bi1—Ba2^iv^	3.5756 (9)
Ba2—Bi1^i^	3.5756 (9)	Bi1—Ba2^i^	3.5756 (9)
Ba2—Bi1^ii^	3.5756 (9)	Bi1—Ba2^ii^	3.5756 (9)
Ba2—Bi1^v^	3.5756 (9)	Bi1—Ba2^v^	3.5756 (9)
Ba2—Cd2^i^	3.613 (9)	Bi2—Cd1^xvi^	2.9688 (14)
Ba2—Cd2^iv^	3.613 (9)	Bi2—Cd1^i^	2.9688 (14)
Ba2—Cd2^ii^	3.613 (9)	Bi2—Cd1^xvii^	2.9688 (14)
Ba2—Cd2^v^	3.613 (9)	Bi2—Cd1^ii^	2.9688 (14)
Ba2—Bi3^vi^	3.6588 (16)	Bi2—Ba1^iv^	3.8575 (16)
Ba2—Bi3^vii^	3.6588 (16)	Bi2—Ba1^i^	3.8575 (16)
Ba2—Bi3^viii^	3.6588 (16)	Bi2—Ba1^v^	3.8575 (16)
Ba2—Bi3^ix^	3.6588 (16)	Bi2—Ba1^ii^	3.8575 (16)
Cd1—Bi2^i^	2.9689 (14)	Bi3—Cd2^xv^	2.771 (13)
Cd1—Bi2^ii^	2.9689 (14)	Bi3—Cd2^x^	2.771 (13)
Cd1—Bi1	3.0565 (14)	Bi3—Cd2^xviii^	2.771 (13)
Cd1—Bi1^x^	3.0565 (14)	Bi3—Bi3^xii^	3.3514 (3)
Cd1—Cd1^xi^	3.3514 (3)	Bi3—Bi3^xi^	3.3514 (3)
Cd1—Cd1^xii^	3.3514 (3)	Bi3—Bi3^xiv^	3.3514 (3)
Cd1—Cd1^xiii^	3.3514 (3)	Bi3—Bi3^xiii^	3.3514 (3)
Cd1—Cd1^xiv^	3.3514 (3)	Bi3—Ba2^i^	3.6588 (16)
Cd1—Ba2^ii^	3.963 (2)	Bi3—Ba2^xix^	3.6588 (16)
Cd1—Ba2^i^	3.963 (2)	Bi3—Ba2^xx^	3.6588 (16)
Cd2—Bi1	2.60 (2)	Bi3—Ba2^ii^	3.6588 (16)
Cd2—Bi3^xi^	2.771 (13)	O—Ba1^i^	2.6736 (14)
Cd2—Bi3^iii^	2.771 (13)	O—Ba1^x^	2.6736 (14)
Cd2—Bi3^xiii^	2.771 (13)	O—Ba1^ii^	2.6736 (14)
Cd2—Bi3	2.771 (13)		
			
O^i^—Ba1—O	77.62 (5)	Bi3^xi^—Cd2—Bi3^iii^	74.4 (4)
O^i^—Ba1—O^ii^	124.84 (12)	Bi1—Cd2—Bi3^xiii^	121.2 (4)
O—Ba1—O^ii^	77.62 (5)	Bi3^xi^—Cd2—Bi3^xiii^	117.5 (9)
O^i^—Ba1—O^iii^	77.62 (5)	Bi3^iii^—Cd2—Bi3^xiii^	74.4 (4)
O—Ba1—O^iii^	124.84 (12)	Bi1—Cd2—Bi3	121.2 (4)
O^ii^—Ba1—O^iii^	77.62 (5)	Bi3^xi^—Cd2—Bi3	74.4 (4)
O^i^—Ba1—Bi2^iv^	140.694 (10)	Bi3^iii^—Cd2—Bi3	117.5 (9)
O—Ba1—Bi2^iv^	140.694 (10)	Bi3^xiii^—Cd2—Bi3	74.4 (4)
O^ii^—Ba1—Bi2^iv^	71.62 (2)	Bi1—Cd2—Cd2^xv^	180.0
O^iii^—Ba1—Bi2^iv^	71.62 (2)	Bi3^xi^—Cd2—Cd2^xv^	58.8 (4)
O^i^—Ba1—Bi2^i^	71.62 (2)	Bi3^iii^—Cd2—Cd2^xv^	58.8 (4)
O—Ba1—Bi2^i^	71.62 (2)	Bi3^xiii^—Cd2—Cd2^xv^	58.8 (4)
O^ii^—Ba1—Bi2^i^	140.694 (10)	Bi3—Cd2—Cd2^xv^	58.8 (4)
O^iii^—Ba1—Bi2^i^	140.694 (10)	Bi1—Cd2—Ba2^i^	68.0 (4)
Bi2^iv^—Ba1—Bi2^i^	120.64 (8)	Bi3^xi^—Cd2—Ba2^i^	138.99 (16)
O^i^—Ba1—Bi2^ii^	140.694 (9)	Bi3^iii^—Cd2—Ba2^i^	138.99 (16)
O—Ba1—Bi2^ii^	71.62 (2)	Bi3^xiii^—Cd2—Ba2^i^	68.47 (4)
O^ii^—Ba1—Bi2^ii^	71.62 (2)	Bi3—Cd2—Ba2^i^	68.47 (4)
O^iii^—Ba1—Bi2^ii^	140.694 (9)	Cd2^xv^—Cd2—Ba2^i^	112.0 (4)
Bi2^iv^—Ba1—Bi2^ii^	75.81 (4)	Bi1—Cd2—Ba2^iv^	68.0 (4)
Bi2^i^—Ba1—Bi2^ii^	75.81 (4)	Bi3^xi^—Cd2—Ba2^iv^	68.47 (4)
O^i^—Ba1—Bi2^v^	71.62 (2)	Bi3^iii^—Cd2—Ba2^iv^	68.47 (4)
O—Ba1—Bi2^v^	140.694 (9)	Bi3^xiii^—Cd2—Ba2^iv^	138.99 (16)
O^ii^—Ba1—Bi2^v^	140.694 (9)	Bi3—Cd2—Ba2^iv^	138.99 (16)
O^iii^—Ba1—Bi2^v^	71.62 (2)	Cd2^xv^—Cd2—Ba2^iv^	112.0 (4)
Bi2^iv^—Ba1—Bi2^v^	75.81 (4)	Ba2^i^—Cd2—Ba2^iv^	136.1 (7)
Bi2^i^—Ba1—Bi2^v^	75.81 (4)	Bi1—Cd2—Ba2^v^	68.0 (4)
Bi2^ii^—Ba1—Bi2^v^	120.64 (8)	Bi3^xi^—Cd2—Ba2^v^	138.99 (16)
O^i^—Ba1—Ba1^i^	38.81 (2)	Bi3^iii^—Cd2—Ba2^v^	68.47 (4)
O—Ba1—Ba1^i^	38.81 (2)	Bi3^xiii^—Cd2—Ba2^v^	68.47 (4)
O^ii^—Ba1—Ba1^i^	103.24 (10)	Bi3—Cd2—Ba2^v^	138.99 (16)
O^iii^—Ba1—Ba1^i^	103.24 (10)	Cd2^xv^—Cd2—Ba2^v^	112.0 (4)
Bi2^iv^—Ba1—Ba1^i^	173.23 (11)	Ba2^i^—Cd2—Ba2^v^	82.0 (3)
Bi2^i^—Ba1—Ba1^i^	66.13 (4)	Ba2^iv^—Cd2—Ba2^v^	82.0 (3)
Bi2^ii^—Ba1—Ba1^i^	107.110 (13)	Bi1—Cd2—Ba2^ii^	68.0 (4)
Bi2^v^—Ba1—Ba1^i^	107.110 (13)	Bi3^xi^—Cd2—Ba2^ii^	68.47 (4)
O^i^—Ba1—Ba1^v^	38.81 (2)	Bi3^iii^—Cd2—Ba2^ii^	138.99 (16)
O—Ba1—Ba1^v^	103.24 (10)	Bi3^xiii^—Cd2—Ba2^ii^	138.99 (16)
O^ii^—Ba1—Ba1^v^	103.24 (10)	Bi3—Cd2—Ba2^ii^	68.47 (4)
O^iii^—Ba1—Ba1^v^	38.81 (2)	Cd2^xv^—Cd2—Ba2^ii^	112.0 (4)
Bi2^iv^—Ba1—Ba1^v^	107.110 (12)	Ba2^i^—Cd2—Ba2^ii^	82.0 (3)
Bi2^i^—Ba1—Ba1^v^	107.110 (13)	Ba2^iv^—Cd2—Ba2^ii^	82.0 (3)
Bi2^ii^—Ba1—Ba1^v^	173.23 (11)	Ba2^v^—Cd2—Ba2^ii^	136.1 (7)
Bi2^v^—Ba1—Ba1^v^	66.13 (3)	Cd2—Bi1—Cd1	129.16 (3)
Ba1^i^—Ba1—Ba1^v^	69.33 (7)	Cd2—Bi1—Cd1^xiii^	129.17 (3)
O^i^—Ba1—Ba1^ii^	103.24 (10)	Cd1—Bi1—Cd1^xiii^	66.49 (4)
O—Ba1—Ba1^ii^	38.81 (2)	Cd2—Bi1—Cd1^xi^	129.17 (3)
O^ii^—Ba1—Ba1^ii^	38.81 (2)	Cd1—Bi1—Cd1^xi^	66.49 (4)
O^iii^—Ba1—Ba1^ii^	103.24 (10)	Cd1^xiii^—Bi1—Cd1^xi^	101.67 (7)
Bi2^iv^—Ba1—Ba1^ii^	107.110 (12)	Cd2—Bi1—Cd1^iii^	129.17 (3)
Bi2^i^—Ba1—Ba1^ii^	107.110 (13)	Cd1—Bi1—Cd1^iii^	101.67 (7)
Bi2^ii^—Ba1—Ba1^ii^	66.13 (3)	Cd1^xiii^—Bi1—Cd1^iii^	66.49 (4)
Bi2^v^—Ba1—Ba1^ii^	173.23 (11)	Cd1^xi^—Bi1—Cd1^iii^	66.49 (4)
Ba1^i^—Ba1—Ba1^ii^	69.33 (7)	Cd2—Bi1—Ba2^iv^	69.60 (4)
Ba1^v^—Ba1—Ba1^ii^	107.10 (14)	Cd1—Bi1—Ba2^iv^	137.22 (3)
O^i^—Ba1—Ba1^iv^	103.24 (10)	Cd1^xiii^—Bi1—Ba2^iv^	137.22 (3)
O—Ba1—Ba1^iv^	103.24 (10)	Cd1^xi^—Bi1—Ba2^iv^	72.92 (3)
O^ii^—Ba1—Ba1^iv^	38.81 (2)	Cd1^iii^—Bi1—Ba2^iv^	72.92 (3)
O^iii^—Ba1—Ba1^iv^	38.81 (2)	Cd2—Bi1—Ba2^i^	69.60 (4)
Bi2^iv^—Ba1—Ba1^iv^	66.13 (3)	Cd1—Bi1—Ba2^i^	72.92 (3)
Bi2^i^—Ba1—Ba1^iv^	173.23 (11)	Cd1^xiii^—Bi1—Ba2^i^	72.92 (3)
Bi2^ii^—Ba1—Ba1^iv^	107.110 (12)	Cd1^xi^—Bi1—Ba2^i^	137.22 (3)
Bi2^v^—Ba1—Ba1^iv^	107.110 (12)	Cd1^iii^—Bi1—Ba2^i^	137.22 (3)
Ba1^i^—Ba1—Ba1^iv^	107.10 (14)	Ba2^iv^—Bi1—Ba2^i^	139.21 (8)
Ba1^v^—Ba1—Ba1^iv^	69.33 (7)	Cd2—Bi1—Ba2^ii^	69.60 (4)
Ba1^ii^—Ba1—Ba1^iv^	69.33 (7)	Cd1—Bi1—Ba2^ii^	72.92 (3)
Bi1^iv^—Ba2—Bi1^i^	139.21 (8)	Cd1^xiii^—Bi1—Ba2^ii^	137.22 (3)
Bi1^iv^—Ba2—Bi1^ii^	83.02 (3)	Cd1^xi^—Bi1—Ba2^ii^	72.92 (3)
Bi1^i^—Ba2—Bi1^ii^	83.02 (3)	Cd1^iii^—Bi1—Ba2^ii^	137.22 (3)
Bi1^iv^—Ba2—Bi1^v^	83.02 (3)	Ba2^iv^—Bi1—Ba2^ii^	83.02 (3)
Bi1^i^—Ba2—Bi1^v^	83.02 (3)	Ba2^i^—Bi1—Ba2^ii^	83.02 (3)
Bi1^ii^—Ba2—Bi1^v^	139.21 (8)	Cd2—Bi1—Ba2^v^	69.60 (4)
Bi1^iv^—Ba2—Cd2^i^	178.4 (4)	Cd1—Bi1—Ba2^v^	137.22 (3)
Bi1^i^—Ba2—Cd2^i^	42.3 (4)	Cd1^xiii^—Bi1—Ba2^v^	72.92 (3)
Bi1^ii^—Ba2—Cd2^i^	97.49 (12)	Cd1^xi^—Bi1—Ba2^v^	137.22 (3)
Bi1^v^—Ba2—Cd2^i^	97.49 (12)	Cd1^iii^—Bi1—Ba2^v^	72.92 (3)
Bi1^iv^—Ba2—Cd2^iv^	42.3 (4)	Ba2^iv^—Bi1—Ba2^v^	83.02 (3)
Bi1^i^—Ba2—Cd2^iv^	178.4 (4)	Ba2^i^—Bi1—Ba2^v^	83.02 (3)
Bi1^ii^—Ba2—Cd2^iv^	97.49 (12)	Ba2^ii^—Bi1—Ba2^v^	139.21 (8)
Bi1^v^—Ba2—Cd2^iv^	97.49 (12)	Cd1^xvi^—Bi2—Cd1^i^	68.73 (4)
Cd2^i^—Ba2—Cd2^iv^	136.1 (7)	Cd1^xvi^—Bi2—Cd1^xvii^	105.92 (7)
Bi1^iv^—Ba2—Cd2^ii^	97.49 (12)	Cd1^i^—Bi2—Cd1^xvii^	68.73 (4)
Bi1^i^—Ba2—Cd2^ii^	97.49 (12)	Cd1^xvi^—Bi2—Cd1^ii^	68.73 (4)
Bi1^ii^—Ba2—Cd2^ii^	42.3 (4)	Cd1^i^—Bi2—Cd1^ii^	105.92 (7)
Bi1^v^—Ba2—Cd2^ii^	178.4 (4)	Cd1^xvii^—Bi2—Cd1^ii^	68.73 (4)
Cd2^i^—Ba2—Cd2^ii^	82.0 (3)	Cd1^xvi^—Bi2—Ba1^iv^	142.059 (16)
Cd2^iv^—Ba2—Cd2^ii^	82.0 (3)	Cd1^i^—Bi2—Ba1^iv^	142.059 (16)
Bi1^iv^—Ba2—Cd2^v^	97.49 (12)	Cd1^xvii^—Bi2—Ba1^iv^	78.92 (4)
Bi1^i^—Ba2—Cd2^v^	97.49 (12)	Cd1^ii^—Bi2—Ba1^iv^	78.92 (4)
Bi1^ii^—Ba2—Cd2^v^	178.4 (4)	Cd1^xvi^—Bi2—Ba1^i^	78.92 (4)
Bi1^v^—Ba2—Cd2^v^	42.3 (4)	Cd1^i^—Bi2—Ba1^i^	78.92 (4)
Cd2^i^—Ba2—Cd2^v^	82.0 (3)	Cd1^xvii^—Bi2—Ba1^i^	142.059 (17)
Cd2^iv^—Ba2—Cd2^v^	82.0 (3)	Cd1^ii^—Bi2—Ba1^i^	142.059 (17)
Cd2^ii^—Ba2—Cd2^v^	136.1 (7)	Ba1^iv^—Bi2—Ba1^i^	120.64 (8)
Bi1^iv^—Ba2—Bi3^vi^	134.01 (4)	Cd1^xvi^—Bi2—Ba1^v^	142.059 (17)
Bi1^i^—Ba2—Bi3^vi^	80.58 (2)	Cd1^i^—Bi2—Ba1^v^	78.92 (4)
Bi1^ii^—Ba2—Bi3^vi^	80.58 (2)	Cd1^xvii^—Bi2—Ba1^v^	78.92 (4)
Bi1^v^—Ba2—Bi3^vi^	134.01 (4)	Cd1^ii^—Bi2—Ba1^v^	142.059 (17)
Cd2^i^—Ba2—Bi3^vi^	44.8 (3)	Ba1^iv^—Bi2—Ba1^v^	75.81 (4)
Cd2^iv^—Ba2—Bi3^vi^	98.0 (3)	Ba1^i^—Bi2—Ba1^v^	75.81 (4)
Cd2^ii^—Ba2—Bi3^vi^	44.8 (3)	Cd1^xvi^—Bi2—Ba1^ii^	78.92 (4)
Cd2^v^—Ba2—Bi3^vi^	98.0 (3)	Cd1^i^—Bi2—Ba1^ii^	142.059 (17)
Bi1^iv^—Ba2—Bi3^vii^	134.01 (4)	Cd1^xvii^—Bi2—Ba1^ii^	142.059 (16)
Bi1^i^—Ba2—Bi3^vii^	80.58 (2)	Cd1^ii^—Bi2—Ba1^ii^	78.92 (4)
Bi1^ii^—Ba2—Bi3^vii^	134.01 (4)	Ba1^iv^—Bi2—Ba1^ii^	75.81 (4)
Bi1^v^—Ba2—Bi3^vii^	80.58 (2)	Ba1^i^—Bi2—Ba1^ii^	75.81 (4)
Cd2^i^—Ba2—Bi3^vii^	44.8 (3)	Ba1^v^—Bi2—Ba1^ii^	120.64 (8)
Cd2^iv^—Ba2—Bi3^vii^	98.0 (3)	Cd2—Bi3—Cd2^xv^	62.5 (9)
Cd2^ii^—Ba2—Bi3^vii^	98.0 (3)	Cd2—Bi3—Cd2^x^	117.5 (9)
Cd2^v^—Ba2—Bi3^vii^	44.8 (3)	Cd2^xv^—Bi3—Cd2^x^	180.0 (9)
Bi3^vi^—Ba2—Bi3^vii^	54.51 (3)	Cd2—Bi3—Cd2^xviii^	179.997 (2)
Bi1^iv^—Ba2—Bi3^viii^	80.58 (2)	Cd2^xv^—Bi3—Cd2^xviii^	117.5 (9)
Bi1^i^—Ba2—Bi3^viii^	134.01 (4)	Cd2^x^—Bi3—Cd2^xviii^	62.5 (9)
Bi1^ii^—Ba2—Bi3^viii^	134.01 (4)	Cd2—Bi3—Bi3^xii^	127.2 (2)
Bi1^v^—Ba2—Bi3^viii^	80.58 (2)	Cd2^xv^—Bi3—Bi3^xii^	127.2 (2)
Cd2^i^—Ba2—Bi3^viii^	98.0 (3)	Cd2^x^—Bi3—Bi3^xii^	52.8 (2)
Cd2^iv^—Ba2—Bi3^viii^	44.8 (3)	Cd2^xviii^—Bi3—Bi3^xii^	52.8 (2)
Cd2^ii^—Ba2—Bi3^viii^	98.0 (3)	Cd2—Bi3—Bi3^xi^	52.8 (2)
Cd2^v^—Ba2—Bi3^viii^	44.8 (3)	Cd2^xv^—Bi3—Bi3^xi^	52.8 (2)
Bi3^vi^—Ba2—Bi3^viii^	80.74 (4)	Cd2^x^—Bi3—Bi3^xi^	127.2 (2)
Bi3^vii^—Ba2—Bi3^viii^	54.51 (3)	Cd2^xviii^—Bi3—Bi3^xi^	127.2 (2)
Bi1^iv^—Ba2—Bi3^ix^	80.58 (2)	Bi3^xii^—Bi3—Bi3^xi^	180.0
Bi1^i^—Ba2—Bi3^ix^	134.01 (4)	Cd2—Bi3—Bi3^xiv^	127.2 (2)
Bi1^ii^—Ba2—Bi3^ix^	80.58 (2)	Cd2^xv^—Bi3—Bi3^xiv^	127.2 (2)
Bi1^v^—Ba2—Bi3^ix^	134.01 (4)	Cd2^x^—Bi3—Bi3^xiv^	52.8 (2)
Cd2^i^—Ba2—Bi3^ix^	98.0 (3)	Cd2^xviii^—Bi3—Bi3^xiv^	52.8 (2)
Cd2^iv^—Ba2—Bi3^ix^	44.8 (3)	Bi3^xii^—Bi3—Bi3^xiv^	90.0
Cd2^ii^—Ba2—Bi3^ix^	44.8 (3)	Bi3^xi^—Bi3—Bi3^xiv^	90.0
Cd2^v^—Ba2—Bi3^ix^	98.0 (3)	Cd2—Bi3—Bi3^xiii^	52.8 (2)
Bi3^vi^—Ba2—Bi3^ix^	54.51 (3)	Cd2^xv^—Bi3—Bi3^xiii^	52.8 (2)
Bi3^vii^—Ba2—Bi3^ix^	80.74 (4)	Cd2^x^—Bi3—Bi3^xiii^	127.2 (2)
Bi3^viii^—Ba2—Bi3^ix^	54.51 (3)	Cd2^xviii^—Bi3—Bi3^xiii^	127.2 (2)
Bi2^i^—Cd1—Bi2^ii^	105.92 (7)	Bi3^xii^—Bi3—Bi3^xiii^	90.0
Bi2^i^—Cd1—Bi1	112.360 (17)	Bi3^xi^—Bi3—Bi3^xiii^	90.0
Bi2^ii^—Cd1—Bi1	112.360 (17)	Bi3^xiv^—Bi3—Bi3^xiii^	180.0
Bi2^i^—Cd1—Bi1^x^	112.360 (17)	Cd2—Bi3—Ba2^i^	66.7 (3)
Bi2^ii^—Cd1—Bi1^x^	112.360 (17)	Cd2^xv^—Bi3—Ba2^i^	113.3 (3)
Bi1—Cd1—Bi1^x^	101.67 (7)	Cd2^x^—Bi3—Ba2^i^	66.7 (3)
Bi2^i^—Cd1—Cd1^xi^	124.361 (18)	Cd2^xviii^—Bi3—Ba2^i^	113.3 (3)
Bi2^ii^—Cd1—Cd1^xi^	55.637 (18)	Bi3^xii^—Bi3—Ba2^i^	62.743 (13)
Bi1—Cd1—Cd1^xi^	56.755 (18)	Bi3^xi^—Bi3—Ba2^i^	117.257 (13)
Bi1^x^—Cd1—Cd1^xi^	123.248 (18)	Bi3^xiv^—Bi3—Ba2^i^	117.257 (13)
Bi2^i^—Cd1—Cd1^xii^	55.636 (18)	Bi3^xiii^—Bi3—Ba2^i^	62.743 (13)
Bi2^ii^—Cd1—Cd1^xii^	124.361 (18)	Cd2—Bi3—Ba2^xix^	113.3 (3)
Bi1—Cd1—Cd1^xii^	123.248 (18)	Cd2^xv^—Bi3—Ba2^xix^	66.7 (3)
Bi1^x^—Cd1—Cd1^xii^	56.755 (18)	Cd2^x^—Bi3—Ba2^xix^	113.3 (3)
Cd1^xi^—Cd1—Cd1^xii^	180.0	Cd2^xviii^—Bi3—Ba2^xix^	66.7 (3)
Bi2^i^—Cd1—Cd1^xiii^	55.637 (18)	Bi3^xii^—Bi3—Ba2^xix^	117.257 (13)
Bi2^ii^—Cd1—Cd1^xiii^	124.361 (18)	Bi3^xi^—Bi3—Ba2^xix^	62.743 (13)
Bi1—Cd1—Cd1^xiii^	56.755 (18)	Bi3^xiv^—Bi3—Ba2^xix^	62.743 (13)
Bi1^x^—Cd1—Cd1^xiii^	123.248 (18)	Bi3^xiii^—Bi3—Ba2^xix^	117.257 (13)
Cd1^xi^—Cd1—Cd1^xiii^	90.0	Ba2^i^—Bi3—Ba2^xix^	180.00 (4)
Cd1^xii^—Cd1—Cd1^xiii^	90.0	Cd2—Bi3—Ba2^xx^	113.3 (3)
Bi2^i^—Cd1—Cd1^xiv^	124.361 (18)	Cd2^xv^—Bi3—Ba2^xx^	66.7 (3)
Bi2^ii^—Cd1—Cd1^xiv^	55.637 (18)	Cd2^x^—Bi3—Ba2^xx^	113.3 (3)
Bi1—Cd1—Cd1^xiv^	123.248 (18)	Cd2^xviii^—Bi3—Ba2^xx^	66.7 (3)
Bi1^x^—Cd1—Cd1^xiv^	56.755 (18)	Bi3^xii^—Bi3—Ba2^xx^	62.743 (13)
Cd1^xi^—Cd1—Cd1^xiv^	90.0	Bi3^xi^—Bi3—Ba2^xx^	117.257 (13)
Cd1^xii^—Cd1—Cd1^xiv^	90.0	Bi3^xiv^—Bi3—Ba2^xx^	117.257 (13)
Cd1^xiii^—Cd1—Cd1^xiv^	180.0	Bi3^xiii^—Bi3—Ba2^xx^	62.743 (13)
Bi2^i^—Cd1—Ba2^ii^	163.77 (5)	Ba2^i^—Bi3—Ba2^xx^	99.26 (4)
Bi2^ii^—Cd1—Ba2^ii^	90.31 (3)	Ba2^xix^—Bi3—Ba2^xx^	80.74 (4)
Bi1—Cd1—Ba2^ii^	59.59 (3)	Cd2—Bi3—Ba2^ii^	66.7 (3)
Bi1^x^—Cd1—Ba2^ii^	59.59 (3)	Cd2^xv^—Bi3—Ba2^ii^	113.3 (3)
Cd1^xi^—Cd1—Ba2^ii^	64.988 (15)	Cd2^x^—Bi3—Ba2^ii^	66.7 (3)
Cd1^xii^—Cd1—Ba2^ii^	115.015 (15)	Cd2^xviii^—Bi3—Ba2^ii^	113.3 (3)
Cd1^xiii^—Cd1—Ba2^ii^	115.015 (15)	Bi3^xii^—Bi3—Ba2^ii^	117.257 (13)
Cd1^xiv^—Cd1—Ba2^ii^	64.988 (15)	Bi3^xi^—Bi3—Ba2^ii^	62.743 (13)
Bi2^i^—Cd1—Ba2^i^	90.31 (3)	Bi3^xiv^—Bi3—Ba2^ii^	62.743 (13)
Bi2^ii^—Cd1—Ba2^i^	163.77 (5)	Bi3^xiii^—Bi3—Ba2^ii^	117.257 (13)
Bi1—Cd1—Ba2^i^	59.59 (3)	Ba2^i^—Bi3—Ba2^ii^	80.74 (4)
Bi1^x^—Cd1—Ba2^i^	59.59 (3)	Ba2^xix^—Bi3—Ba2^ii^	99.26 (4)
Cd1^xi^—Cd1—Ba2^i^	115.015 (15)	Ba2^xx^—Bi3—Ba2^ii^	180.00 (4)
Cd1^xii^—Cd1—Ba2^i^	64.988 (15)	Ba1—O—Ba1^i^	102.38 (5)
Cd1^xiii^—Cd1—Ba2^i^	64.988 (15)	Ba1—O—Ba1^x^	124.84 (12)
Cd1^xiv^—Cd1—Ba2^i^	115.015 (15)	Ba1^i^—O—Ba1^x^	102.38 (5)
Ba2^ii^—Cd1—Ba2^i^	73.45 (5)	Ba1—O—Ba1^ii^	102.38 (5)
Bi1—Cd2—Bi3^xi^	121.2 (4)	Ba1^i^—O—Ba1^ii^	124.84 (12)
Bi1—Cd2—Bi3^iii^	121.2 (4)	Ba1^x^—O—Ba1^ii^	102.38 (5)

Symmetry codes: (i) −*x*+1/2, −*y*+1/2, −*z*+1/2; (ii) −*x*−1/2, −*y*+1/2, −*z*+1/2; (iii) *x*, *y*−1, *z*; (iv) −*x*−1/2, −*y*−1/2, −*z*+1/2; (v) −*x*+1/2, −*y*−1/2, −*z*+1/2; (vi) −*y*+1/2, *x*+1/2, *z*+1/2; (vii) *x*+1/2, *y*−1/2, *z*+1/2; (viii) −*y*+1/2, *x*−1/2, *z*+1/2; (ix) *x*−1/2, *y*−1/2, *z*+1/2; (x) *x*, *y*+1, *z*; (xi) −*y*, *x*, *z*; (xii) −*y*+1, *x*+1, *z*; (xiii) −*y*+1, *x*, *z*; (xiv) −*y*, *x*+1, *z*; (xv) −*x*, −*y*, −*z*; (xvi) *y*−1/2, −*x*+1/2, −*z*+1/2; (xvii) *y*−1/2, −*x*−1/2, −*z*+1/2; (xviii) −*x*, −*y*+1, −*z*; (xix) *x*−1/2, *y*+1/2, *z*−1/2; (xx) *x*+1/2, *y*+1/2, *z*−1/2.

## References

[bb1] Brechtel, E., Cordier, G. & Schäfer, H. (1981). *J. Less Common Met.* **79**, 131–136.

[bb2] Bruker (2002). *SMART*, *SAINT* and *SADABS* Bruker AXS Inc., Madison, Wisconsin, USA.

[bb3] Cordier, G., Woll, P. & Schäfer, H. (1982). *J. Less Common Met.* **86**, 129–134.

[bb4] *CrystalMaker* (2009). *CrystalMaker* CrystalMaker Software Ltd, Bicester, England.

[bb5] Gelato, L. M. & Parthé, E. (1987). *J. Appl. Cryst.* **20**, 139–143.

[bb6] Papoian, G. A. & Hoffmann, R. (2000). *Angew. Chem. Int. Ed.* **39**, 2408–2448. 10.1002/1521-3773(20000717)39:14<2408::aid-anie2408>3.0.co;2-u 10941096

[bb7] Saparov, B. & Bobev, S. (2010). *Dalton Trans.* doi:10.1039/c0dt00595a. 10.1039/c0dt00595a 20944855

[bb8] Saparov, B., Xia, S.-Q. & Bobev, S. (2008). *Inorg. Chem.* **47**, 11237–11244. 10.1021/ic801530d 19228027

[bb14] Sheldrick, G. M. (2008). *Acta Cryst* A**64**, 112–122. 10.1107/S0108767307043930 18156677

[bb9] Villars, P. & Calvert, L. D. (1991). *Pearson’s Handbook of Crystallographic Data for Intermetallic Compounds*, 2nd ed. Materials Park, Ohio, USA: American Society for Metals.

[bb10] Xia, S.-Q. & Bobev, S. (2006*a*). *Inorg. Chem.* **45**, 7126–7132. 10.1021/ic060583z 16933913

[bb11] Xia, S.-Q. & Bobev, S. (2006*b*). *J. Solid State Chem.* **179**, 3371–3377.

[bb12] Xia, S.-Q. & Bobev, S. (2007). *Chem. Asian J.* **2**, 619–624. 10.1002/asia.200700016 17457792

[bb13] Xia, S.-Q. & Bobev, S. (2008). *Inorg. Chem.* **47**, 1919–1921. 10.1021/ic800242a 18288797

